# Patterns, trends and determinants of medical opioid utilization in Canada 2005–2020: characterizing an era of intensive rise and fall

**DOI:** 10.1186/s13011-021-00396-5

**Published:** 2021-09-14

**Authors:** Wayne Jones, Ridhwana Kaoser, Benedikt Fischer

**Affiliations:** 1 Centre for Applied Research in Mental Health and Addiction (CARMHA), Faculty of Health Sciences, Simon Fraser University, Suite 2400, 515 W. Hastings Street, British Columbia Vancouver, Canada; 2 Faculty of Medical and Health Sciences, University of Auckland, 85 Park Road, Auckland 1023 Grafton, New Zealand; 3 Department of Psychiatry, University of Toronto, 250 College Street, Toronto, Ontario Canada; 4 Department of Psychiatry, Federal University of São Paulo (UNIFESP), R. Sena Madureira, 1500 - Vila Clementino, São Paulo, Brazil

**Keywords:** Opioids, Pharmacoepidemiology, Canada, Pain, Utilization, Policy

## Abstract

**Background:**

Into the 21st century, the conflation of high rates of chronic pain, systemic gaps in treatment availability and access, and the arrival of potent new opioid medications (e.g., slow-release oxycodone) facilitated strong increases in medical opioid dispensing in Canada. These persisted until post-2010 alongside rising opioid-related adverse (e.g., morbidity/mortality) outcomes. We examine patterns, trends and determinants of opioid dispensing in Canada, and specifically its 10 provinces, for the years 2005–2020.

**Methods:**

Raw data on prescription opioid dispensing were obtained from a large national community-based pharmacy database (IQVIA/Compuscript), converted into Defined-Daily-Doses/1,000 population/day for ‘strong’ and ‘weak’ opioid categories per standard methods. Dispensing by opioid category and formulations by province/year was assessed descriptively; regression analysis was applied to examine possible segmentation of over-time strong opioid dispensing.

**Results:**

All provinces reported starkly increasing strong opioid dispensing peaking 2011–2016, and subsequent marked declines. About half reported lower strong opioid dispensing in 2020 compared to 2005, with continuous inter-provincial differences of > 100 %; weak opioids also declined post-2011/12. Segmented regression suggests breakpoints for strong opioids in 2011/12 and 2015/16, coinciding with main interventions (e.g., selective opioid delisting, new prescribing guidelines) towards more restrictive opioid utilization control.

**Conclusions:**

We characterized an era of marked rise and fall, while featuring stark inter-provincial heterogeneity in opioid dispensing in Canada. While little evidence for improvements in pain care outcomes exists, the starkly inverting opioid utilization have been associated with extensive population-level harms (e.g., misuse, morbidity, mortality) over-time. This national case study raises fundamental questions for opioid-related health policy and practice.

## Introduction

The transition into the 21st century marked a turning point for chronic pain and related opioid pharmacotherapy in North America, and Canada specifically. There, 20–29 % of the gcomes in the populationeneral population were estimated to experience chronic pain in the early 2000s, however systemic gaps existed in access to and/or effectiveness of available care [1, 2]. More broadly, a vocal socio-medical movement had begun to propagate chronic pain as a neglected ‘fifth vital sign’, advocating for systematic expansions of improved treatment and care [3, 4], including more generous utilization of opioid pharmacotherapy options then generally considered “safe and efficacious” [1]. Among other barriers, pharmacotherapeutic approaches to chronic pain care were viewed as hindered by many physicians’ lack of knowledge and/or hesitation to prescribe available opioid medications for chronic pain [5, 6].

A number of concrete developments facilitated a profound expansion of opioid pharmacotherapy for chronic pain in the early 2000s in Canada. Sequential iterations of national pain care guidelines (updated in 2002) recommended a wider-scale and more generous (e.g., higher doses/long-term) therapeutic utilization of opioid analgesic medications for chronic pain [7]. In addition, new and potent opioid formulations – most notably including ‘slow-release’ oxycodone (e.g., Oxycontin) – had become available and became widely utilized, alongside other potent semi-/synthetic opioid medications (e.g., fentanyl, hydromorphone) that were increasingly and more generously prescribed in both specialist and general/community practice settings [8–10]. Particularly in the case of Oxycontin, their rising utilization was distinctly boosted by pharmaceutical companies’ targeted marketing strategies involving ‘peer educators’ (i.e., physician representatives) promoting expanded prescribing [11, 12].

In these contexts, the population rate of controlled total opioid dispensing in Canada - as reported by the International Narcotics Control Board (INCB) - more than tripled within just one decade, from 8,713 Standardized Defined Daily Doses [S-DDD] in 2000–2002 to 29,743 S-DDD in 2010-12 [13]. During this period, Canada’s opioid dispensing rate climbed more steeply than that of the United States (US), the nation featuring the world’s highest opioid consumption rate; on this basis, Canada rose to record the world’s second-highest opioid consumption rate (after the US) [14]. By 2010, more than one-in-five (20 %) of Canadian adults reported annual use of pain-related opioid medications [15].

While the benefits for therapeutic pain care quality and outcomes from these extensive increases have remained uncertain, they were associated with rising adverse opioid-related (e.g., mortality and morbidity) outcomes in the population, including increases in non-medical opioid use and diversion, opioid-related hospitalizations, treatment admissions and poisoning fatalities [15–17]. For example, in Ontario, 6 % of adults and 15 % of high-school students reported non-medical opioid use by 2010, opioid-related treatment admissions doubled and opioid-related fatalities rose from 366 (2003) to 571 (2010), with approximately 40 % oxycodone-related [15, 18, 19]. By 2010, it was evident that a sort of ‘opioid crisis’ involving extensive adverse health outcomes in the population -- fuelled by the excessive availability and adverse consequences of prescription opioids -- was unfolding in Canada [8, 20, 21].

While government and other (e.g., medical/regulatory) policy makers and stakeholders had undertaken little to halt the growing opioid-related harms until then, a series of multi-level interventions aiming to restrict opioid availability and harms were implemented post-2010 [22]. Among them, slow-release oxycodone formulations were delisted from most provincial formularies across Canada in 2012 [23, 24]. Several provinces introduced (e.g., Ontario in 2012) or ramped up their ‘prescription monitoring’ systems including opioids in their scope [25, 26]. A national stakeholder coalition tabled a strategic action plan of proposed measures [2013] to reduce opioid-related harms [27]. Select regulatory efforts aimed to increase the use of ‘safer’ (e.g., tamper-resistant/abuse-deterrent) opioid formulations, and to limit high-dose prescribing [23, 28]. Meanwhile, emerging scientific evidence assumed an increasingly cautious and restrained view on the effectiveness and safety of opioid pharmacotherapy especially for chronic pain care [29, 30]. Correspondingly, new Canadian opioid prescribing guidelines (2017), similar to recent US (CDC) counterparts launched in 2016, presented a marked reversal from previous guideline versions and provided direction for a generally much more restrained and cautious (‘last resort’) approach to opioid pharmacotherapy utilization and practice for pain care [31].

Following these interventions, on overall decline in medical opioid utilization began to unfold. Canada’s total opioid utilization peaked at 34,444 S-DDD in 2013-15, and substantively decreased to 19,629 S-DDD (-43 %) by 2017-19 [13]. While prescription opioid-related harms (e.g., poisoning fatalities) remained generally steady, expanding availability and use of illicit/synthetic opioid (e.g., fentanyl) products started to shift and accelerate patterns especially of non-medical opioid harms in the years since 2014/15 across Canada [32–34]. In 2018, the federal government established of the Canadian Pain Task Force, with a mandate to assess the state of pain and related care practices and systems in Canada [35].

Within these wider contexts, and building on previous related examinations, the present study assesses overall (quantitative & qualitative) patterns, trends and determinants of medical opioid utilization in Canada and specifically its 10 provinces for the period 2005–2020.

## Methods

The primary data on prescription opioid dispensing in Canada for the period 2005–2020 were derived and computed based on information from the IQVIA Canada Inc. (formerly QuintilesIMS/IMS Brogan) Compuscript database. This database monitors prescription-based transactions for branded and generic medications via a representative and stratified sample of about 6,500 (representing about 60 % of the total) retail pharmacies across Canada [36, 37]. Monthly dispensing data is aggregated to provincial totals using a patented geospatial projection methodology, with an estimated sampling error of 5–10 %. The Compuscript data do not include non-prescription (e.g., ‘over-the-counter’ codeine) products, nor cover other (e.g., hospital-based) drug dispensing, yet comprise the far majority (estimated 80 %+) of total utilization. Similar dispensing data have been used for other population-level pharmacoepidemiologic analyses [38, 39].

Based on previously applied methods, raw prescription opioid dispensing information was obtained by yearly totals for the 10 Canadian provinces (but not including the three Canadian territories, which however only make up < 0.5 % of the Canadian total population) of both the numbers of prescriptions and units dispensed, opioid molecule (codeine, fentanyl, hydrocodone, hydromorphone, meperidine, methadone, morphine, oxycodone, tramadol), and product name (494 unique names included), form and strength information. Data for the different opioid products were matched to defined daily dose (DDD) estimates using the World Health Organization’s Anatomical Therapeutic Chemical classification and DDD measurement methodology, defining DDDs as the “… assumed average maintenance dose per day for a drug used for its main indication in adults” [40]. DDD are a standard metric commonly used for comparative drug utilization analysis [41, 42]. Combined with yearly provincial population estimates (obtained from Statistics Canada [43]) the total opioid dispensing data was converted to annual DDDs/per 1,000 population/per day (DDD/1,000/day) estimates for the 10 provinces and Canada total, and furthermore categorized into “strong opioids” (i.e., including fentanyl, hydrocodone, hydromorphone, meperidine, morphine, and oxycodone) and “weak opioids” (i.e., codeine) generally following the WHO ‘analgesic ladder’ [44, 45]. Methadone, while defined as a strong opioid, was excluded from the analyses since it is primarily used for addiction (i.e., opioid maintenance) treatment, and its dispensing practices are inconsistent and do not allow for comparable estimates across Canada [37].

Database design and data manipulation was conducted using the R software package, including data plotting for visualization [46]. For descriptive analyses, first, we examined the annual dispensing levels of “strong” (including main individual formulations) and “weak” (codeine) opioid products for Canada and the provinces, 2005–2020. Second, we computed the intra-provincial ranges of highest and lowest annual strong opioid dispensing values for the study period. Third, towards assessing significant over-time changes in strong opioid dispensing in Canada, we applied a segmented (or ‘broken stick’) regression analysis [47]. This regression-based method partitions the independent variable into intervals and fits corresponding straight-lines to data interval-subsets (here the 16 annual datapoints of strong opioid dispensing), while identifying possible inter-segmental ‘breakpoints’ [48, 49]. Akaike information criterion (AIC) values were computed to assess the quality-of-fit for each model. For these analyses, the R package ‘segmented’ was used.

## Results

### Strong opioids (total) [see Fig. 1 for data visualization]

**Fig. 1:**
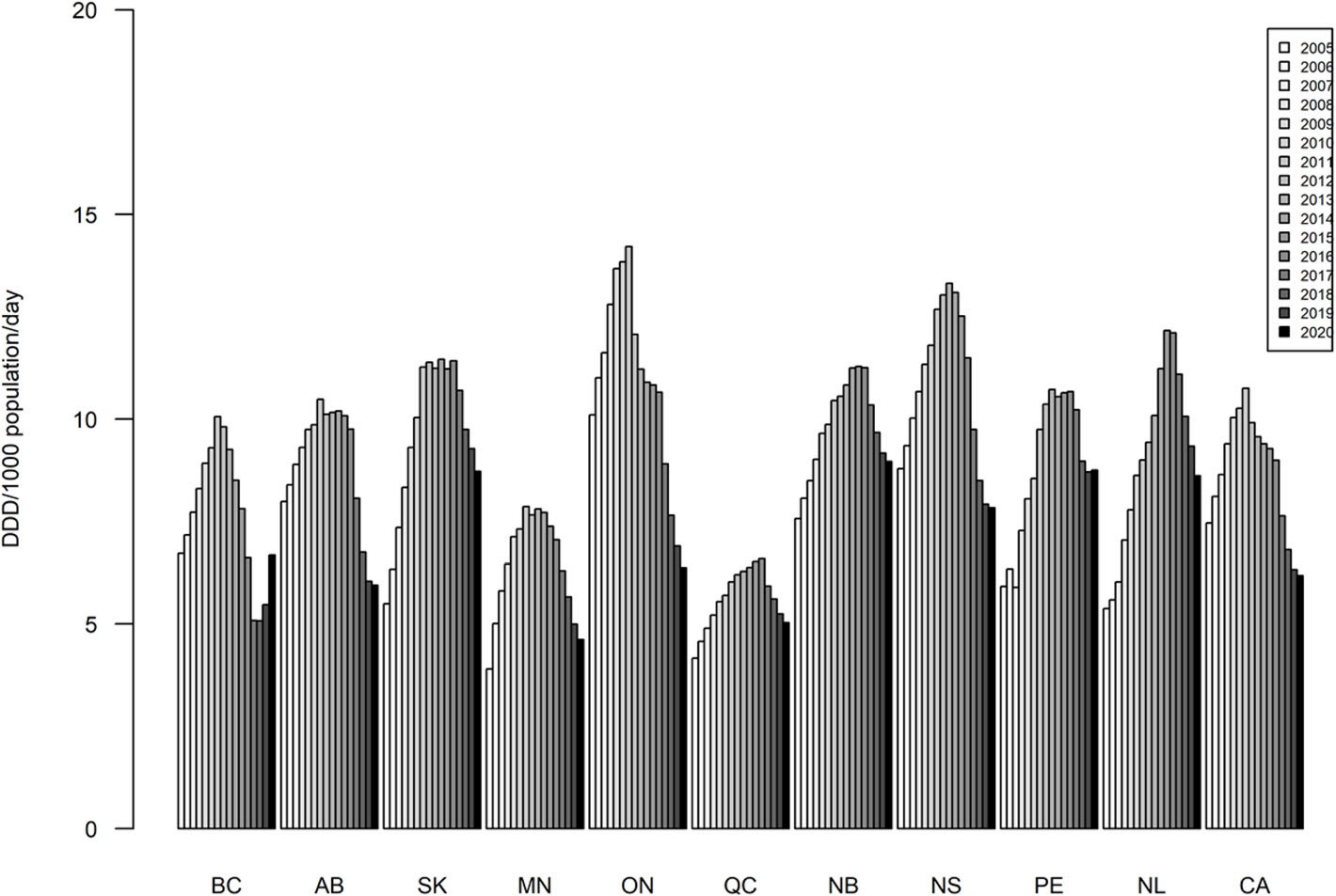
Annual ‘strong opioid’ utilization in DDD/1,000 population/day by province and Canada total, 2005–2020

In 2005, the lowest strong opioid dispensing rate was reported by Manitoba (MN;3.9 DDD/1,000/day), and the highest by Ontario (ON;10.1 DDD/1,000/day), translating into an inter-provincial range of difference of 159 %.

Between 2005 and 2011, each of the provinces reported substantive increases in their annual rates of strong opioid dispensing, with individual provinces’ respective opioid dispensing levels peaking sometime between 2011 and 2016 as follows: British Columbia (BC), Alberta (AB), MN, ON (2011); Nova Scotia (NS), Prince Edward Island (PEI;2013); Saskatchewan (SK;2014); New Brunswick (NB), Newfoundland (NL;2015); and Quebec (QC;2016). The highest ‘peak’ value for opioid dispensing was reported by ON (14.2 DDD/1,000/day), and the lowest ‘peak’ value by QC (6.6 DDD/1,000/day), indicating a total inter-provincial range of difference of 115 % for ‘peak’ levels.

Following their respective ‘peak’ level years, each of the provinces reported decreases in strong opioid dispensing, declining to - with the exception of two provinces (BC & PEI) – lowest post-peak level of strong opioid dispensing in 2020. ON reported the proportionally greatest decrease between its respective ‘peak’ level year in strong opioid dispensing (2011) and 2020 (-54.9 %) whereas PEI (2016–2020) had the correspondingly smallest decrease (-17.8 %). In 2020, the lowest opioid dispensing rate was report by MN (4.6 DDD/1,000/day), and the highest rate by NB (9.0 DDD/1,000/day), translating into an inter-provincial range of difference of 96 %. Four provinces reported same, or lower strong opioid dispensing levels in 2020 compared with 2005.

For intra-provincial variation in annual strong opioid dispensing, NB indicated the smallest range (49 %) and NL indicated the largest range (126 %) over the observation period (2005–2020) (see Table 1).

**Table 1:** Intra-provincial ranges and percent differences in ‘strong opioid’ utilization, provinces and Canada total, 2005–2020

	DDD/1000/Day: Years 2005–2020		
Province	Minimum	Maximum	Difference (%)
BC	5.1	10.1	98.3 %
AB	5.9	10.5	76.2 %
SK	5.5	11.5	108.8 %
MN	3.9	7.9	101.7 %
ON	6.4	14.2	123.2 %
QC	4.2	6.6	58.3 %
NB	7.6	11.3	49.2 %
NS	7.8	13.3	69.9 %
PEI	5.9	10.7	82.0 %
NL	5.4	12.2	126.1 %
CA	6.2	10.8	74.1 %

Abbreviations: *BC *British Columbia, *AB *Alberta, *SK *Saskatchewan, *MN *Manitoba, *ON *Ontario, *QC *Quebec, *NB * New Brunswick, *NS *Nova Scotia, *PEI *Prince Edward Island, *NL *Newfoundland, *CA *Canada

### Strong opioids (individual formulations) [see Fig. 2 for data visualization]

**Fig. 2:**
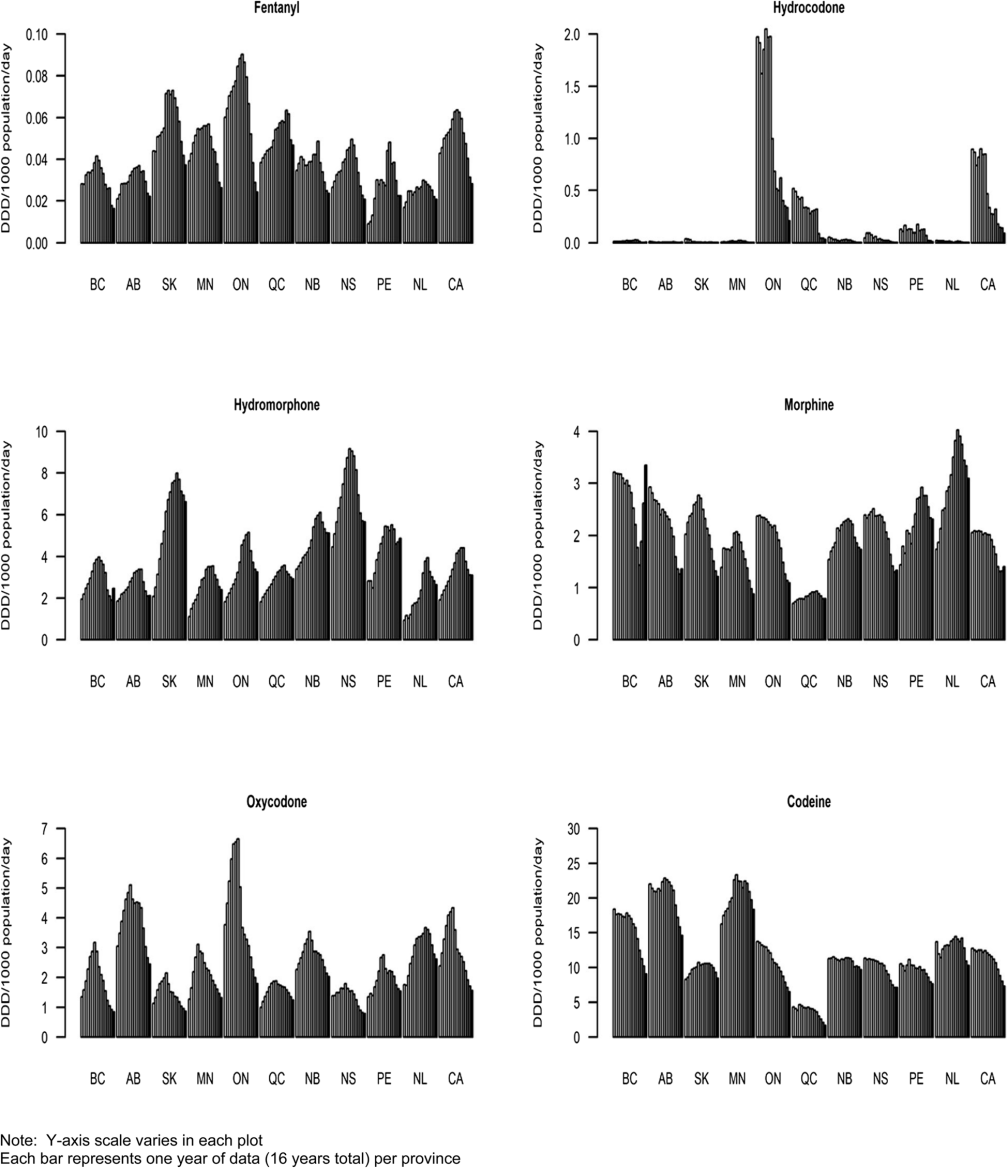
Annual utilization of individual opioid formulations in DDD/1,000 population/day by province and Canada total, 2005–2020

For main individual strong opioid formulations, varying trends and patterns were observed by province. For *fentanyl*, all provinces reported increases in dispensing until 2014/2015 which then inverted to varying levels of decreases. In any given year, the inter-provincial levels of fentanyl dispensing varied by at least 100 %; half the provinces reported lower fentanyl dispensing rates in 2020 compared to 2005. *Hydrocodone* has been dispensed almost exclusively in ON and, at much lower levels, in QC; its utilization in ON was stable until 2011, and then steeply dropped. *Hydromorphone* (similar to fentanyl) dispensing increased in all provinces to 2015/2016, and subsequently inverted to decrease. While in any given year, hydromorphone dispensing levels inter-provincially varied by at least 100 %, each province reported higher hydromorphone dispensing in 2020 as compared with 2005. While some provinces reported increases in *morphine* dispensing in the early years of observation, other reported mostly decreasing trends; half the provinces indicated lower morphine dispensing levels in 2020 compared with 2005. BC is a noted outlier for both hydromorphone and morphine dispensing, in that it features marked recent increases in dispensing for both recent opioid drugs; its morphine utilization in 2020 is the highest for the total study period. For *oxycodone*, most provinces reported strong dispensing increases (most of them peaking in 2011) which subsequently inverted to substantive decreases. In each year, the inter-provincial dispensing range for oxycodone differed by at least 200 %, while the majority of provinces reported lower oxycodone dispensing levels in 2020 compared with 2005.

### Weak opioids [see Fig. 2 for data visualization]

The majority of provinces reported relatively steady levels in weak opioid (codeine) dispensing with most peak levels in 2011/2012; subsequently, weak opioid dispensing declined in all provinces, and each province except one (SK) reported lower weak opioid dispensing in 2020 compared with 2005. In each year, the inter-provincial range of weak opioid dispensing rates differed by at least 300 %.

### Multilinear (segmented regression) analysis [see Fig. 3 for results visualization]

**Fig. 3:**
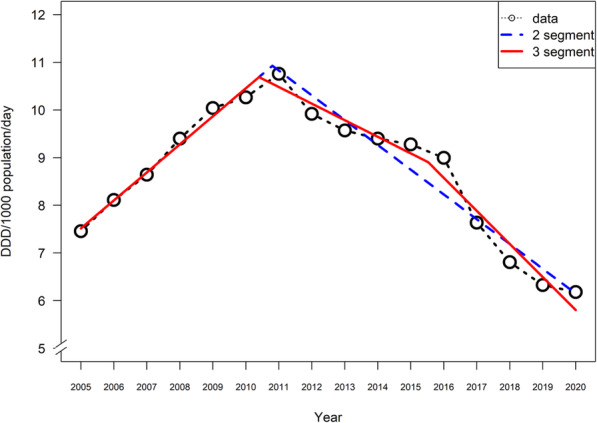
Two- and three-segment models of ‘strong opioid’ utilization in Canada, 2005–2020

Visual inspection of the over-time pattern for strong opioid dispensing (see above) for Canada total suggests an overall non-linear inversion shape, yet it is unclear whether this may involve additional segments. On this basis, the segmented regression analysis confirmed a strictly linear model to be not statistically significant (adjusted R-square: -0.05). A four-segment model (using possible breakpoints at 2008, 2013, and 2017) failed to converge. A three-segment model (breakpoints: 2010 and 2016) was statistically significant (adjusted R-square: 0.96), based on an increasing linear segment running from 2005 to 2010/11, a decreasing linear segment from 2010/11 to 2015/16, and then a steeper decreasing linear segment from 2015/16 to 2020. A two-segment model (breakpoint: 2012) was also statistically significant (R-square: 0.94), based on an increasing linear segment from 2005 to 2011, followed by a decreasing linear segment from 2011 to 2020. Of the models examined, the three-segment model fit the observed data best with an AIC value of 12.4, followed by the two-segment model (AIC: 17.0).

## Discussion

We characterized quantitative and qualitative patterns and trends in medical opioid dispensing in Canada, with focus on its ten provinces, for the period 2005–2020. These analyses – extending previous Canada-based examinations - are worthwhile towards assessing both the evolution and drivers of medical opioid utilization in Canada as appear to represent and complete a distinct ‘era’, yet also more broadly given Canada’s status as a global high-consumer nation [13, 50, 51].

The first, main observation is that over the study period, Canada underwent a marked bi-partial evolution – concretely a stark increase-to-decrease reversal pattern - of strong opioid utilization. In aggregate, its strong opioid utilization initially increased by almost 50 % (2005–2011/12), and subsequently decreased by a similar proportion (2011/12–2020) to an overall lower level compared to 2005. In other words, the overall Canada-wide population exposure for opioids first increased, and then decreased by about half its total volume (in DDD), within just a few years. Such a pronounced rise-and-fall development in the system-wide utilization of controlled psychotropic medications, or any medical intervention geared to a chronic disease (here mainly: pain) with overall stable prevalence in the population ought to be considered highly unusual, if not exceptional. These developments naturally seek for analytic understanding and contextualization of both the essential drivers behind this starkly bi-directional pattern as well as their impacts.

Our analyses begin at a point-in-time (2005) following both increasing national and international attention to ‘chronic pain’ as a prevalent, but inadequately treated health condition [1, 52–54]. These contexts, jointly with the emergence of new medical practice guidelines advocating for more liberal and generous opioid pharmacotherapy use especially in the context of pain care, and the rapidly expanding availability of new and potent opioid medications (e.g., slow-release oxycodone) evidently facilitated a substantive increase in medical opioid prescribing and expanding population exposure in Canada within just a few years [7, 8, 51]. Yet, by the overall ‘peak’ in opioid utilization (2011/2012) it became both increasingly evident that the safety and efficacy of strong opioids widely used for pain-based pharmacotherapy care had been mis-assessed, and that the extensive increases in strong opioid prescribing were associated with extensive collateral harms in the population [8, 21, 55]. Concretely, mounting evidence indicated substantive increases in opioid-related non-medical use, morbidity (e.g., hospitalizations) and mortality (e.g., poisoning deaths) in Canada; for several of the main adverse outcome indicators, strong correlations with population-levels of prescription opioid dispensing were statistically confirmed, meaning that these harm outcomes increased in direct correlation with the changing volumes of opioids available in the population [15, 56, 57].

As suggested by the results of our ‘segmented regression’ analyses, the timepoint of the pan-Canadian delisting of slow-release oxycodone (‘Oxycontin’) from provincial formularies (2012) as an initial, system-wide intervention to reduce strong opioid availability marked an initial major inversion point towards decreasing strong opioid dispensing [23, 24]. This intervention resulted in some lateral shifts in specific types of strong opioids prescribed (e.g., from oxycodone to hydromorphone, fentanyl) yet appears to have triggered an overall system-wide change and reduction effect in opioid utilization. This particular intervention was further complemented - depending on provincial setting or opioid drug type - by a range of additional (many provincially-based) restricting measures especially for high-risk opioid utilization in subsequent years (e.g., prescription monitoring program expansion, limitations on high-dose formulations) as selectively assessed for their deceleration impact, creating an aggregately more restrictive opioid prescribing environment in Canada [22, 26, 50, 58–60].

The second ‘breakpoint’ identified, around 2016/17, may be identified to coincide with the lead-up to and the introduction of new Canadian guidelines for chronic pain treatment tabled in 2017; these new guidelines followed a corresponding set of new US-based guidelines presented just somewhat earlier by the CDC [2016], which already had received substantial attention and select regulatory uptake in Canadian jurisdictions (e.g., BC) with demonstrated reduction effects on opioid prescribing [31, 61–63]. The new Canada-based guidelines conveyed a paradigmatically different spirit and message from their predecessors, advising towards a generally restrained and cautious, largely ‘last resort’ approach for the utilization of strong opioid pharmacotherapy in the context of pain care [64–67].

The stark inversion developments in strong opioid utilization observed in Canada, to a substantial extent, mirror developments in the US, where rising and exceptionally high levels of strong opioid dispensing reverted to decrease following a combination intensified regulatory, monitoring and enforcement interventions [68–70]. Specifically, the implementation of the new CDC opioid prescribing guideline (2016) has been assessed to be associated with significant decreases in opioid prescribing in the US [71, 72]. While Canada traditionally has also been a high-consumer country for codeine (‘weak’) opioid products, the observed utilization patterns somewhat follow those for strong opioids. Their utilization, somewhat similarly, also begins to decline around 2010/11, albeit without formal interventions but a rather increasingly restrictive awareness and climate in the medical-scientific community emphasizing their limited efficacy and safety, and the subsequent need for reductions in utilization or for banning use altogether [73–75].

Overall, the observed reversing patterns especially in levels of strong opioid dispensing within just a few years in Canada are remarkable, but also reflect the starkly evolving, or even contradictory evidence that have been informing and guiding related medical views and practices during this time. While available scientific literature considered strong opioids as generally ‘effective and safe’ for pain therapy in the early 2000s, and their use was systematically promoted by pharmaceutical producers and prescribers alike on this basis, subsequently evolving evidence increasingly underlined limitations and risks [30, 76, 77]. Another influencing factor towards change outside formal policy or regulatory measures involves wider socio-cultural forces, for example, mass media or investigative reports that have focused on the emerging opioid crisis and its facets in Canada post-2012 [78]. These likely also contributed to changing broader public awareness and vigilance, and professional practices in regards to opioids and their use.

Further noteworthy based on the pharmaco-epidemiologic data presented is the degree of heterogeneity specifically of province-based patterns and trends in strong opioid dispensing in Canada over time. In any given year, the range of inter-provincially highest and lowest strong opioid dispensing levels reaches a difference of (nearly) 100 %. Of note, many - while not all - of the ‘lows’ in strong opioid dispensing are recorded by Quebec, Canada’s socio-culturally distinct francophone province, whereas many ‘highs’ occurred in Ontario (Quebec’s directly adjacent neighbor, and Canada’s most populous province). Moreover, the patterns of strong opioid dispensing in several – especially smaller Eastern – provinces inverted to decreasing trends only with a few years’ delay. While Canada is a confederation where matters of health regulation and practice are predominantly controlled by the provinces, such stark contrasts within the same nation providing for principles of universal healthcare are worth underscoring [79]. Therefore, the determinants of these inter-provincial differences may include pronvincially-based regulatory system factors and differences (e.g., as related to provincial drug formularies, medical practice regulations, prescription drug monitoring etc.) but may also involve more ‘soft’ factors like medical practice culture, norms or training [25, 80–82]. The drivers between these distinct intra-Canadian heterogeneities in opioid utilization should be more systematically examined by appropriate methods and data.

The starkly contrasting patterns of opioid utilization in Canada observed raise basic questions in regards to their – direct and indirect - impacts and effects on relevant health outcomes. While the initial increases in opioid utilization were driven by high rates of chronic pain and related care system deficiencies, there is discernably little concrete evidence that the substantive expansions in opioid utilization through the early 2000s has led to concrete improvements in pain care quality or outcomes [17, 83–85]. The recently established (2018) ‘Canadian Pain Task Force assesses a continuously unfavourable and inadequate picture of the state of pain and related care – characterizing it as “a public health emergency in need of action” as recently as 2021 - that appears little different from the situation observed in the early 2000s [35]. Conversely, various (mostly qualitative/local) studies have documented how current chronic pain patients receiving opioid pharmacotherapy have been forced to taper off their opioid medications, or experienced increasing barriers towards opioid medications access in contexts of recent, increasingly restrictive opioid control environments [86–88]. Thus, the overall benefits of the extensive expansions, and subsequent contraction of strong opioid availability as a distinct chapter in health policy practice in Canada appear to be marked by fundamental questions.

Moreover, the substantive un-intended adverse consequences from extensively high population-level opioid exposure, including high rates of non-medical opioid use, opioid-related morbidity (hospitalizations, treatment admissions) and especially mortality (poisoning fatalities) including adverse impacts on population life expectancy have entailed an exceptional burden of adverse outcomes [15, 34, 89–92]. Most recently, Canada’s opioid-related poisoning death rate rapidly rose from 7.8/100,000 (2016) to 17.0/100,000 (2020), translating to a total of 6,214 opioid-related fatalities in 2020 and indicating similar rates to those recorded in the US [34, 93]. While the dramatic increases in fatalities have been attributed mostly to illicit/synthetic opioids, some analyses suggest that these drugs proliferated partly in response to ‘supply shocks/gaps’ following the rapid and substantive reductions in prescription opioid supply available for non-medical use despite persistent demand [33, 94, 95]. On this basis, the policy ‘case study’ of the marked inversion of strong opioid utilization in Canada, as documented, have come with distinctly mixed, including considerable adverse impacts relevant for overall population health [96, 97].

Further to the data presented, BC notably represents an outlier province for recent developments in opioid dispensing, in that it features marked re-increases in strong opioid (i.e., morphine, hydromorphone) utilization in very recent (2018–2020) years. These increases appear mainly driven by a growing number of ‘safer opioid supply’ programs recently initiated in BC (Vancouver) that provide individuals involved with high-risk opioid (e.g., illicit fentanyl or analogues) exposure with less toxic, prescription-grade strong opioid supply (e.g., hydromorphone, sustained-release morphine pills) as a public health measure towards reducing the acute risk for opioid overdose and death [98–100]. On this basis, somewhat ironically, strong opioid dispensing and availability has been re-expanding mainly to remedy the adverse public health consequences – directly or indirectly - facilitated by previous over-exposure and subsequent rapid restrictions in pharmaceutical opioid supply [94, 95, 97]. It also deserves mention that our data extend to 2020, the first year of the COVID-19 pandemic, which has distinctly influenced psychoactive substance use and may have influenced prescription opioid utilization patterns in different while uncertain ways in that particular year. Specifically, there may have been increased demand for or use of prescription opioids (e.g., for pain or other adverse symptoms) due to COVID-19 related circumstances, yet simultaneously increased access barriers and practice changes to health care provision may have reduced medication use [101–103].

The present study and analyses include several limitations. The community-pharmacy sample-based data used for opioid dispensing calculation does not capture other dispensing points (e.g., hospitals, internet pharmacies), and so may involve underestimations. DDD as a comparative opioid measurement metric is limited in reliability, but similar in its limitations to that of other measures (e.g., morphine equivalents) and superior to crude indicators like numbers of prescriptions [42, 104]. The segmented regression may be limited by the limited number of data years available for the analyses. Moreover, segmented regression fits linear segments while it is possible that in some cases non-linear segments could provide a better fit for the data, a possibility not examined here. In addition, results can be influenced by the choice of initial values but this is often mitigated by basing the selection on examination of initial scatter plots [48]. The population-level dispensing data do not include the three Canadian territories, and so formally do not represent the Canada ‘total’; however, the territories only include < 0.5 % of the total Canadian population. Analyses for Canada totals of opioid dispensing are population-weighted, and so are dominated by trends in the more populous (e.g., ON) provinces.

## Conclusions

We have characterized an era of intensive rise-and-fall in opioid dispensing, while featuring marked inter-provincial heterogeneity, in Canada over the (brief) period 2005–2020. This case study raises fundamental questions for health policy making, and specifically opioid medications utilization and control in contexts of pain care, psychotropic medications use and public health for Canada, but beyond for other jurisdictions aiming to develop appropriate and effective health policy approaches in these realms. Essentially, Canada in regards to opioid utilization, after a pronounced period of ‘up-and-down’, finds itself back where it was in the early 2000s, yet without substantive evidence for marked improvements in pain care quality or outcomes while recording extensive adverse effects for public health from extensive population-level opioid exposure. The stark oscillation developments observed, and related consequences experienced over the past 20 years naturally cannot be corrected or reversed now. Looking forward, it will be essential for Canada – within the complexities of its health care and policy systems - to find a more stable and evidence-informed state or equilibrium that better balances evidence-based pain care and related clinical opioid utilization needs for those individuals who require them, while preventing undue opioid exposure and reducing related adverse consequences for the general population and the benefit of public health. To some extent, other industrialized nations (e.g., select European countries or New Zealand) have taken more restrained and cautious approaches to medical opioid utilization and – even in contexts of increases in utilization - have experienced overall lower levels of related harms, and therefore may offer useful case studies or guiding evidence to Canada or other jurisdictions in these respects going forward [105–107].

## Data Availability

The raw data on prescription opioid dispensing in Canada were commercially obtained IQVIA Solutions Canada Inc., with all subsequent data processing and analyses solely conducted by the authors. Population data statistics used for the analyses were obtained from Statistics Canada. The external data sources/providers had no involvement or influence in any steps of the analysis development, results generation or data interpretation, or other aspects of the manuscript.

## Abbreviations

DDD: Defined Daily Doses
US: United States
DDD: Defined Daily Doses
BC: British Columbia
AB: Alberta
SK: Saskatchewan
MN: Manitoba
ON: Ontario
QC: Quebec
NB: New Brunswick
NS: Nova Scotia
PE: Prince Edward Island
NL: Newfoundland and Labrador
CA: Canada
AIC: Akaike information criterion
CDC: Center for Disease Control
